# Bronchogenic cyst or lung cancer. Only biopsy can tell

**DOI:** 10.1016/j.rmcr.2020.101328

**Published:** 2020-12-31

**Authors:** Paul Zarogoulidis, Kosmas Tsakiridis, Anastasios Vagionas, Wolfgang Hohenforst-Schmidt, Bojan Zaric, Stavros Tryfon, Maria Saroglou, Konstantinos Drevelegas, Dimitrios Hatzibougias, Electra Michalopoulou-Manoloutsiou, Dimitris Paliouras, Nikolaos Barbetakis, Haidong Huang, Chong Bai

**Affiliations:** a 3rd University General Hospital, ‘‘AHEPA’’ University Hospital, Thessaloniki, Greece; b Thoracic Surgery Department, ‘‘Interbalkan’’ European Medical Center, Thessaloniki, Greece; c Oncology Department, General Hospital of Kavala, Kavala, Greece; d Sana Clinic Group Franken, Department of Cardiology / Pulmonology / Intensive Care / Nephrology, “Hof” Clinics, University of Erlangen, Hof, Germany; e Institute for Pulmonary Diseases of Vojvodina, Faculty of Medicine, University of Novi Sad, Serbia; f Pulmonary Department (NHS), ‘‘G. Papanikolaou’’ General Hospital, Thessaloniki, Greece; g Radiology Department, ‘‘Euromedica’’ Private Radiology Laboratory, Thessaloniki, Greece; h Private Pathology Laboratory, ‘‘Microdiagnostics’’, Thessaloniki, Greece; i Thoracic Surgery Department, ‘‘Theageneio’’ Cancer Hospital, Thessaloniki, Greece; j Department of Respiratory Medicine, Changhai Hospital of Second Military Medical University, Shanghai, China

**Keywords:** Lung cancer, Bronchogenic cyst, EBUS, 19G needle

## Abstract

Bronchogenic cysts are rare congenital malformations which derive from primitive ventral foregut. They are usually observed in intrathoracically. A fifty year old male was admitted for the investigation of a three month chest pain. Computed tomography scan of the thorax revealed a lesion around the esophagus and left stem bronchus. Endobronchial ultrasound with convex probe and a 19G needle biopsy revealed a bronchogenic cystic which was removed with video assisted thoracic surgery. Initial radiologic assessment although was thought to be lung cancer because of the smoking habit it turned out to be benignancy. EBUS-TBNAB with 10G needle is safe and absolutely necessary for these lesions, as they take large samples.

## 1 Introduction

Bronchogenic cysts are rarely found in the everyday clinical practice and they are usually congenital lesions from aberrant budding of the embryonic foregut [1]. They may occur at any region of the mediastinum, however; they have been reported in other sites of the body such as; neck [2], ileal mesentery [3], intradiafragmatic [4], intrapericardial [5], suprasellar [6] and larynx [7]. They are usually observed in males and there are reports-studies where they account for the 10% of mediastinum lesions [8]. The symptoms vary depending the location and size upon diagnosis and during the cyst growth such as; chronic cough, dysphagia, dyspnea, hoarseness, chest pain and increased stridor during sleeping. Upon diagnosis careful assessment should be made for each patient, in order to avoid a lung cancer diagnosis or other malignancy. Endobronchial ultrasound is an effective minimal invasive diagnostic tool for initial evaluation [[9], [10], [11], [12]].

## 2 Case report

**Fig. 1:**
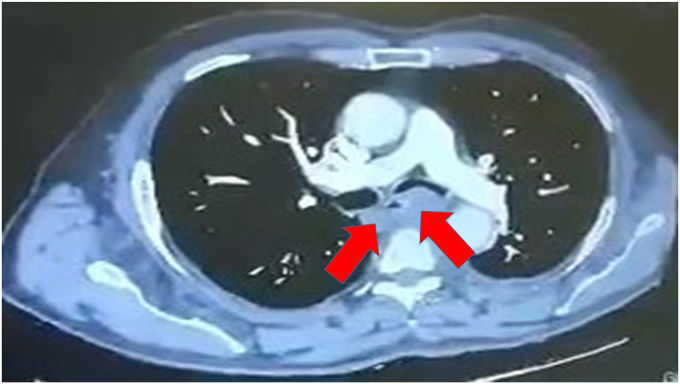
Red arrows indicate the bronchial cyst.

**Fig. 2:**
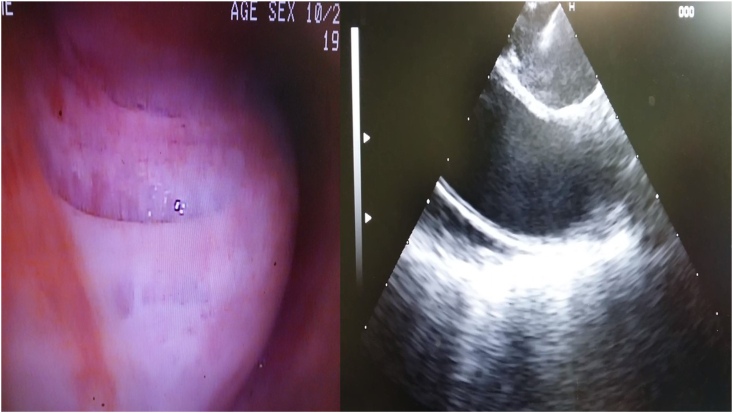
From left to right: Left; is the endoscopic view of the left stem bronchus, right; is the endosonography performed from the EBUS-TBNAB 19G needle puncture.

**Fig. 3:**
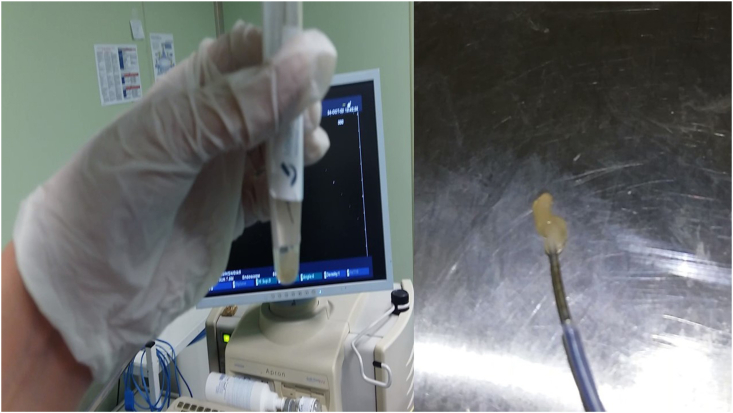
From left to right: Left; is a tube with cytolyte with the biopsy material, Right; is the material that came out in one of the biopsies.

**Fig. 4:**
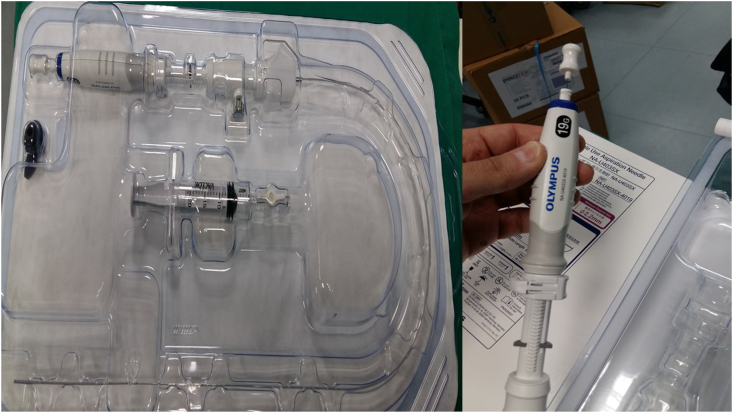
19G Olympus® needle that was used.

**Fig. 5:**
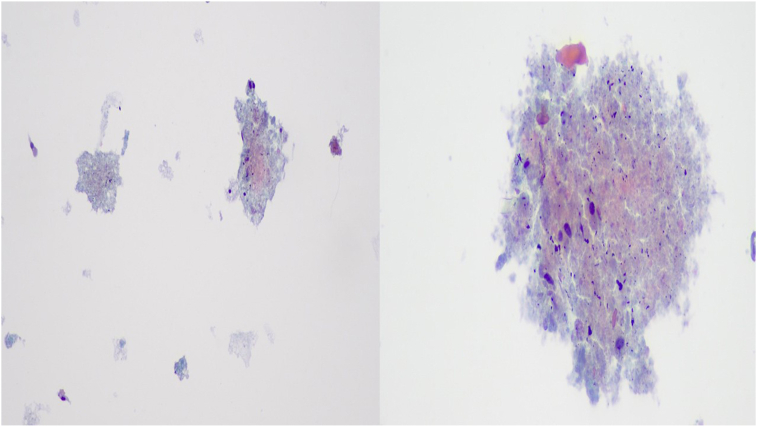
The Papanicolaou stain on the Liquid-Based Cytology specimen (ThinPrep), revealed amorphous material, scattered inflammatory cells, a few pulmonary macrophages, and fringed cylindrical cells of the bronchial epithelium (magnification X400).

**Fig. 6:**
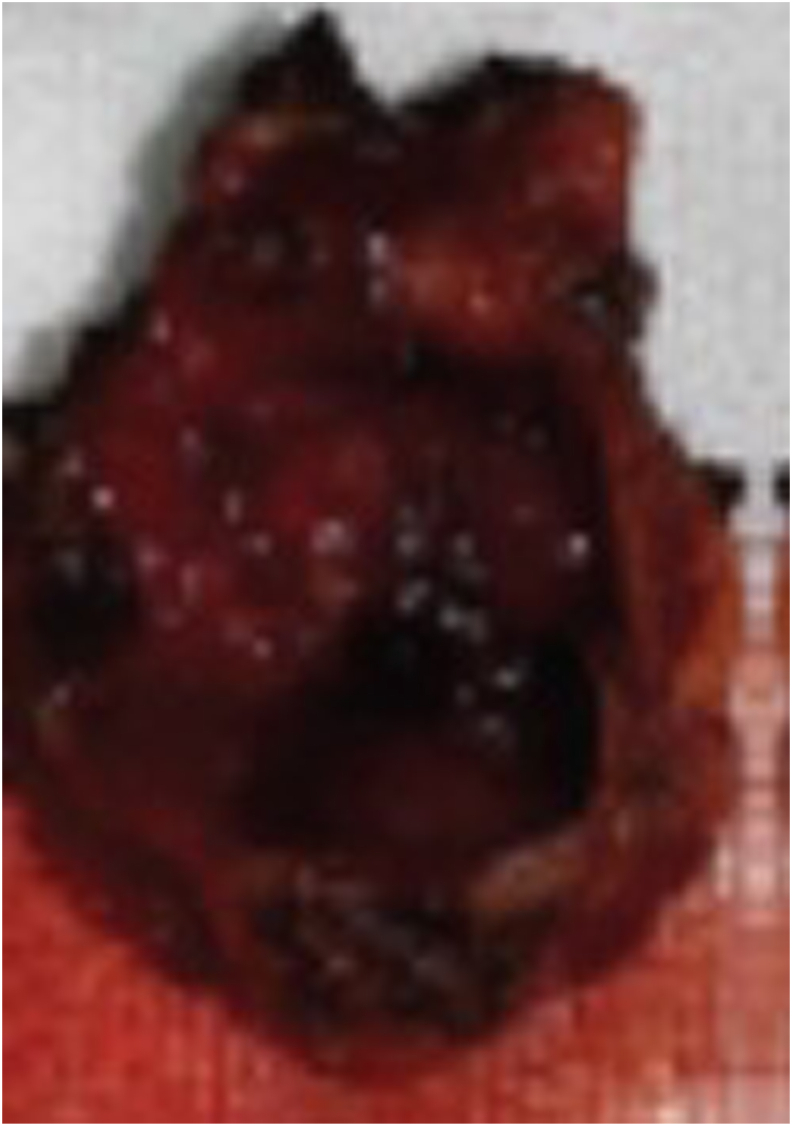
The cyst after video assisted surgery.

**Fig. 7:**
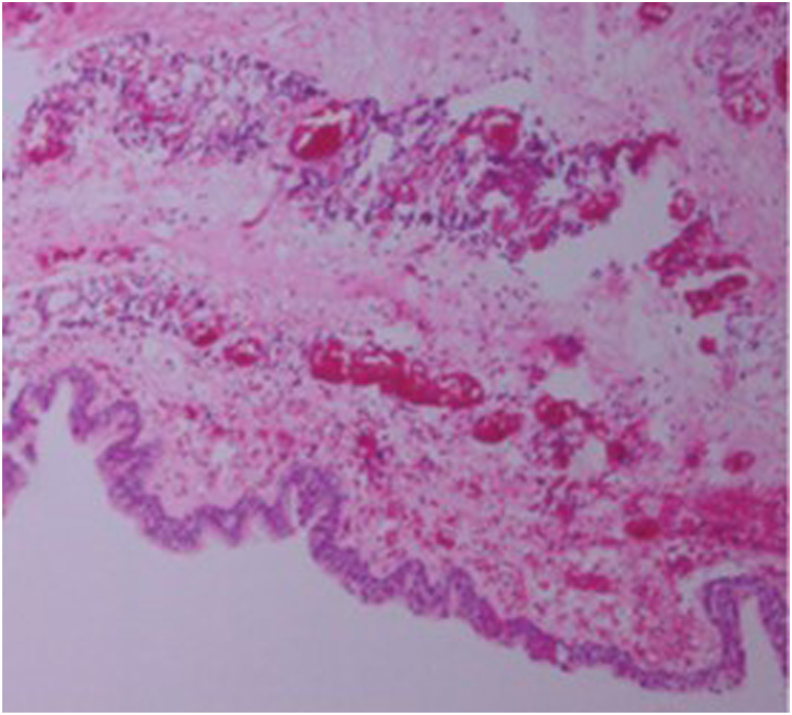
Paraffin-embedded tissue slice cut to 3 μm sections and stained with hematoxylin-eosin-saffron microscope (magnification, x100).

A fifty year old man was admitted for chest pain. In specific the pain had a direction from left to the right finishing in the vertebra in the middle of the thorax. The man had the chest pain for three months, but he chose to take pain relief tablets, until he came to the hospital to be evaluated. All tests were negative for an acute ischemic event. A CT of the thorax with contrast revealed a lesion around the esophagus and the wall of the left stem bronchus (Fig. 1). The patient underwent EBUS-TBNAB 19G with a PENTAX system EPK-1000 and HITACHI EUB 7000UK. There was no lesion inside the left stem bronchus, however; there was obstruction from the lesion growing in the outside wall of the bronchus (Fig. 2). Biopsy was performed with a 19G Oympus® needle and a ‘‘slime’’ yellowish material was obtained with four sequential punctures (Fig. 3, Fig. 4). The material obtained with the 19G needle can be seen in Fig. 5. We used jet-Ventilation for the EBUS-TBNAB procedure [13]. The patient underwent video assisted thoracic surgery (VATS) and the pathological specimen can be found in Fig. 6. The tissue specimen from the VATS surgery can be found at Fig. 7.

## 3 Discussion

Lesions observed in the thorax are usually malignancies and positron emission tomography (PET-CT) can assist in providing us useful information before we perform a biopsy. Usually such lesions do not have F-18 fluorodeoxyglucose (FDG) uptake. However; this might not be true in all cases since false positive uptake could be observed if inflammation exists [1]. Moreover; if the patient has smoking habit then malignancy should be definitely ruled out. The only method to do so is by performing a minimal invasive biopsy. Endobronhial ultrasound with convex probe and 19G needle can provide us with sufficient material in order to have the first evaluation before surgery can be performed. We can perform a minimal invasive surgery with VATS in the case were the lesion is inside the thoracic cavity as in our case and other reported cases [1,14]. We report the first case with EBUS-TBNAB 19G needle. The location of the lesion excluded a thymic cyst and the surgery excluded an esophagus cyst. Moreover; lymphoma was also excluded with the VATS tissue specimen. The main issue that we have to be careful is not to infect the cyst with microbes from the respiratory tract, therefore a fast surgery should be performed as in our case after the biopsy and biopsy result. We have to avoid the case of mediastinitis. Furthermore the VATS surgery will provide us with better tissue sample and avoid lymphoma or bronchogenic carcinoma - pulmonary melioidosis [15]. Usually these lesions have low echogenity, however; in our case since the material inside the cyst was stiff as this is observed from the CT of the thorax, EBUS ECHO picture and needle puncture (Fig. 3) [16]. Management is based on the symptoms, in the case of lack of symptoms then observation should be the best option. In the case of high risk patients for surgery, then aspiration (if there is low echogenity) is an option. However; the best case scenario for symptomatic patients that can undergo surgery VATS is the best option for lesions inside the thoracic cavity [16]. The housefield units and EBUS CONVEX probe can be used to efficiently assess these lesions. Surgery should be the best therapeutic option since infection, hemorage or cancer can occur within the cyst [16].

## Declaration of competing interest

None to declare.
